# Retraction: The protective effect of PPARγ in sepsis-induced acute lung injury via inhibiting PTEN/β-catenin pathway

**DOI:** 10.1042/BSR-20192639_RET

**Published:** 2021-04-19

**Keywords:** acute lung injury, apoptosis, inflammation, PPARγ, PTEN/β-catenin pathway, sepsis

This Retraction follows an Expression of Concern relating to this article previously published by Portland Press.

This article is being retracted from *Bioscience Reports* at the request of the authors following receipt of a notification from a reader, alerting the authors to duplications in Figures 2C, 5A and 5C.

The authors are unable to correct the article and wish to retract the article. The Editor-in-Chief and Editorial Board agree with the retraction.

